# Simultaneous Quantification of Multiple Bacteria by the BactoChip Microarray Designed to Target Species-Specific Marker Genes

**DOI:** 10.1371/journal.pone.0055764

**Published:** 2013-02-11

**Authors:** Annalisa Ballarini, Nicola Segata, Curtis Huttenhower, Olivier Jousson

**Affiliations:** 1 Centre for Integrative Biology, University of Trento, Trento, Italy; 2 Department of Biostatistics, Harvard University, Boston, Massachusetts, United States of America; University of North Carolina at Charlotte, United States of America

## Abstract

Bacteria are ubiquitous throughout the environment, the most abundant inhabitants of the healthy human microbiome, and causal pathogens in a variety of diseases. Their identification in disease is often an essential step in rapid diagnosis and targeted intervention, particularly in clinical settings. At present, clinical bacterial detection and discrimination is primarily culture-based, requiring both time and microbiological expertise, especially for bacteria that are not easily cultivated. Higher-throughput molecular methods based on PCR amplification or, recently, microarrays are reaching the clinic as well. However, these methods are currently restricted to a small set of microbes or based on conserved phylogenetic markers such as the 16S rRNA gene, which are difficult to resolve at the species or strain levels. Here, we designed and experimentally validated the BactoChip, an oligonucleotide microarray for bacterial detection and quantification. The chip allows the culture-independent identification of bacterial species, also determining their relative abundances in complex communities as occur in the commensal microbiota or in clinical settings. The microarray successfully distinguished among bacterial species from 21 different genera using 60-mer probes targeting a novel set of *in silico* identified high-resolution marker genes. The BactoChip additionally proved accurate in determining species-level relative abundances over a 100-fold dynamic range in complex bacterial communities and with a low limit of detection (0.1%). In combination with the continually increasing number of sequenced bacterial genomes, future iterations of the technology could enable to highly accurate clinically-oriented tools for rapid assessment of bacterial community composition and relative abundances.

## Introduction

Modern molecular and metagenomic methods have given new life to the problem of microbial detection, either singly or in communities, and it remains a challenge in a wide variety of contexts: pathogen detection in the clinic [1], [2], surveys both of host-associated microbiota [3], [4] and of environmental communities [5], and microbial forensics [6], to name only a few. Bacteria represent the majority of most such microbial communities [3], [7], but detecting specific bacteria can present a challenge; culture-based methods are applicable to only a small fraction of bacteria [8], and sequence-based methods targeting marker genes such as the 16S rRNA often lack phylogenetic resolution [9], [10]. This raises further issues, as many microbes of interest including *Staphylococcus aureus* [11], *Escherichia coli* [12], or *Mycobacterium* spp. [13] have a pathogenic potential only at the species or strain level. An opportunity thus exists for methods bridging the cost-effectiveness and specificity of culture-based microbial quantification with the breadth and throughput of modern sequence-based techniques. Bacterial culture typically identifies microbial clades within a sample based on phenotypic (morphological or biochemical) features [14], [15]. The earliest methods of bacterial species detection were culture-based, and these techniques continue to dominate clinical screens to this day [16]. While these techniques have been highly optimized for a selection of common organisms of interest, they are difficult to generalize. They can be applied only to bacteria which are cultivable *in vitro*, and in cases where no pre-existing standard protocol is available, optimizing new culture conditions can be time-consuming and laborious to derive [17].

A variety of molecular methods are now also employed in clinical laboratories, which can in many cases elucidate microbial community composition at a much higher resolution than culture-based methods [18]. These include real-time quantitative PCR (qPCR), multiplex PCR, microarray and high-throughput sequence based techniques. Both qPCR and multiplex-PCR represent rapid and simple methods to detect a small panel of target bacteria in a complex mixture by amplification of species-specific DNA regions. However, even multiplex PCR typically scales only to detection of dozens of microbes simultaneously, and clinical PCR-based tests are prone to amplification biases, false positive detections, and erroneous quantification of relative abundances [19]. Application of these methods to quantify many bacteria in a complex mixture would thus require a broad panel of qPCR probes, drastically increasing the costs and time required for the analysis.

Conversely, although high-throughput sequencing techniques for microbial identification have as yet to reach the clinic, they are being employed in metagenomic research [20]. Like PCR, they often target specific taxonomic marker genes, usually the 16S rRNA gene [20], [21]. Amplification and sequencing of such genes from a complex sample using “universal” primers can simultaneously quantify most bacteria in a community, although again with phylogenetic resolution limited to approximately the genus level [21]. Most recently, shotgun metagenomic sequencing has become practical for surveying an entire community’s metagenomic [22], but this is neither a practical method for simple microbial quantification nor yet feasible in a clinical setting [23].

Phylogenetically targeted microarrays have been successfully proposed as a means to bridge the gap between throughput, cost, and clinical feasibility [24]. In contrast to PCR and DNA sequencing methods, the microarray format for microbial community characterization permits the quantification of a broad panel of microbes maintaining low cost per sample. Some microbe detection arrays developed to date have been designed with short probes (e.g. 25-mers) tiled to perfectly match target genes flexibly spanning multiple species of interest (e.g. the prototype Respiratory Pathogen Microarray RPM v1.1 [25]). Chips adopting short oligonucleotide probes may guarantee almost no cross-hybridization events, but present a lower sensitivity [26], thus requiring a pre-amplification step of the microbial DNA and again precluding quantification.

Microbe and microbial function detection arrays designed with longer probes (e.g. 60-mer) have been applied both for environmental microbes (e.g. PhyloChip [27], GeoChip [28]) and for host-associated bacteria (e.g. GreenChip [29], HOMIM [18], HITChip [7]). These arrays all currently target a combination of known functional classes genes or, most commonly, variants of the single 16S rRNA gene. Importantly, longer probes for the taxonomically well-studied 16S rRNA gene typically show higher sensitivity than short probes, but this incurs low resolution due to cross-hybridization of similar sequences across taxa. This has been specifically reported to cause over-estimation of community richness [30], since the highly conserved 16S rRNA gene often fails to discriminate below the genus level for many clades [24]. Taxonomic identification with such chips can therefore require complex bioinformatic postprocessing to avoid copy number or cross-hybridization biases, and even in the best case the species- or strain-level resolution needed for pathogen detection is precluded.

We have thus designed the BactoChip to simultaneously address issues of sensitivity, specificity, and cost in microbial detection microarrays, aiming to render them feasible for very high-throughput, low cost-per-sample microbiome studies in human cohorts. First, we designed long probes targeting clade-specific marker genes by assembling a catalog of markers unique to 54 bacteria of medical interest drawn from all available microbial genomes. We then experimentally validated a custom oligonucleotide microarray using 57- to 60-mer hybridization probes targeting these genes. The array, termed the BactoChip, showed high performance in identifying the presence of single organisms at the species level, even among mixtures of multiple species from the same genus. Additionally, it proved able to quantify the relative abundances of these species in mixed communities over a 100-fold range and with a limit of detection below 0.1% in abundance. The Bactochip thus provides the potential to detect and quantify microbial species and strains with higher accuracy and throughput than available methods, particularly as future revisions of the chip scale to include additional sequenced genomes. The sensitivity of detection demonstrated by this study may prove applicable both for rapid, cost-effective detection in the clinic and for further investigations of the human microbiome and other complex microbial communities.

## Results

### Design of Species-specific Marker Probes for the BactoChip

**Figure 1:**
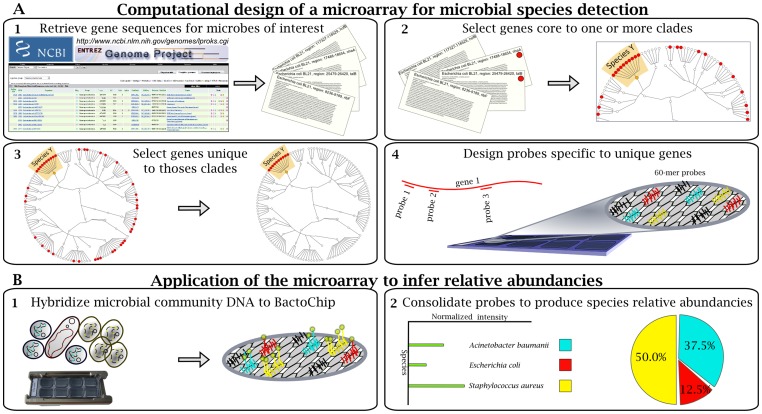
Design of the BactoChip and its application for microbial species identification and quantification. (A) Schematic overview of computational design and its output. (A1) Complete genomes from 186 bacterial species were retrieved from the National Centre for Biotechnology Information microbial database. (A2) Gene sequences core to each target species were defined on the basis of sequence conservation within each clade. Red dots represent the distribution of core genes shared by strains within and outside a target clade. (A3) Core genes unique to each target species were selected by sequence alignment against all available archaeal and bacterial sequences. (A4) Oligonucleotide probes were designed for up to 10 identified unique genes for each target bacterial species. Each probe color represents specificity to a defined bacterial species. (B) Experimental design and example data. (B1) DNA from microbial communities was tested on the BactoChip. Green dots represent Cy3 bound to genomic DNA fragments from a sample hybridized to the chip. (B2) Species relative abundances are finally inferred by normalization of the fluorescence signal for each probe and species.

We developed an oligonucleotide microarray to simultaneously identify a broad range of bacterial species with clinical relevance to human health. In contrast to current phylogenetic microarray approaches for microbial identification, which target individual universally conserved genes such as the 16S rRNA, we identified *in silico* those genes that most unequivocally characterized each bacterial species. Unique, conserved regions within these genes were subsequently targeted for microarray probe design (Figure 1).

Specifically, for the 37 experimentally tested species reported in Table 1 (of 54 total included in the initial probe design), we identified nucleotide-coding sequences strongly conserved (≥99% identity in most cases) among genomes within each species (see Methods). Such genes were considered “core” for each species; we subsequently identified the subset of core genes without substantial similarity (no local matches with e-value lower than 1e-5) to any genomic sequence outside the target species, making them specific to that species. The initially small number of targeted species was limited by available genome sequence and strain accessibility at the time of chip design, and the bioinformatic approach employed is applicable to any broader set of sequenced bacterial genomes. The first step in design thus considers all genomes available for each species of interest, whereas the latter employs all sequenced bacterial and archaeal genomes. When selecting among candidate genes for microarray probe design, unique genes with the greatest copy numbers and sequence lengths were prioritized, in order to maximize detection sensitivity and facilitate probe subsequence selection, respectively. This step resulted in 340 genes being targeted from among 37 testable species chosen from 900 available finished genomes. Hybridization probes were designed for biophysical consistency and compatibility by means of the Allele ID software (Premier Biosoft International, Palo Alto, CA, USA), which accounts for hybridization efficiency and avoids potential cross-hybridization. Our microarray design is provided at the Array Express Repository with accession number A-MEXP-2138, and data for all arrays, run during these experiments, is provided with accession E-MEXP-3420.

**Table 1:** BactoChip specifications for the 19 target genera used in this study.

Target Genera	Target Species	Average (SD) genes/species	Min – Max genes/species	Average (SD) probes/gene	Min – Max probes/gene
*Acinetobacter*	*A. baumanii*	9.0 (0.0)	9–9	1.0 (0.0)	1–1
*Bacteroides*	*B. fragilis, B. thetaiotaomicron, B. vulgatus*	9.0 (0.8)	8–10	1.9 (1.7)	1–6
*Citrobacter*	*C. koseri, C. rodentium*	8.0 (0.0)	8–8	2.4 (1.7)	1–6
*Clostridium*	*C. difficile, C. kluyveri, C. perfringens*	6.7 (1.7)	5–9	1.8 (1.4)	1–5
*Corynebacterium*	*C. diphteriae, C. efficiens, C. jeikeium*	8.0 (0.8)	7–9	2.4 (1.6)	1–6
*Enterococcus*	*E. faecalis*	7.0 (0.0)	7–7	3.0 (1.9)	1–6
*Escherichia*	*E. coli, E. fergusonii*	5.0 (2.0)	3–7	2.0 (1.1)	1–4
*Haemophilus*	*H. influenzae*	9.0 (0.0)	9–9	1.0 (0.0)	1–1
*Klebsiella*	*K. pneumoniae*	2.0 (0.0)	2–2	1.0 (0.0)	1–1
*Listeria*	*L. innocua, L. monocytogenes*	7.0 (3.0)	4–10	1.9 (1.5)	1–6
*Micrococcus*	*M. luteus*	6.0 (0.0)	6–6	2.0 (1.2)	1–4
*Ochrobactrum*	*O. anthropi*	8.0 (0.0)	8–8	2.5 (1.4)	1–5
*Propionibacterium*	*P. acnes*	6.0 (0.0)	6–6	3.8 (2.0)	1–6
*Proteus*	*P. mirabilis*	7.0 (0.0)	7–7	2.6 (1.7)	1–5
*Pseudomonas*	*P. aeruginosa, P. fluorescens, P. mendocina, P. putida*	4.3 (2.9)	2–9	1.6 (1.2)	1–5
*Salmonella*	*S. enterica*	9.0 (0.0)	9–9	2.3 (0.9)	1–4
*Staphylococcus*	*S. aureus, S. carnosus, S- epidermidis, S. haemolyticus, S. lugdunensis, S. saprophyticus*	7.3 (3.0)	1–10	2.4 (1.9)	1–6
*Stenotrophomonas*	*S. maltophilia*	4.0 (0.0)	4–4	1.0 (0.0)	1–1
*Streptococcus*	*S. equi, S. pyogenes*	9.0 (1.0)	8–10	1.1 (0.3)	1–2

The 19 tested target genera are listed here, together with a list of the species tested for each genus and the corresponding average number of target genes per species and probes per gene tested in this study with the BactoChip.

The number of species-specific markers varied considerably across the bacterial kingdom**,** reflecting the phylogenetic diversity of each species, their genome sizes, pan- versus core genomes, and degree of functional specialization [31]. For example, the high similarity of the *Escherichia* and *Shigella* genera limited the number of genes distinguishing individual species to at most 50. *Staphylococcus* species, on the other hand, were instead more diverged, with a minimum within the genus of 281 markers for *S. aureus* and a maximum of 847 for *S. saprophyticus*. The highest numbers of unique species-characterizing genes were detected for *Bacteroides*, with 1,236, 1,702, and 1,952 genes for *B. fragilis*, *B. vulgatus*, and *B. thetaiotaomicron*, respectively. Among more than 30,000 unique marker genes identified for the 54 bacterial species during design (see Materials and methods and ArrayExpress database, experiment E-MEXP-3420), 581 markers were selected and targeted for microarray probes, resulting in a total of 2,094 57/60-mer probes (with three technical replicates each per chip), an average of 116±140 probes for each species, and 3.6±2.7 distinct probes for each marker gene. 520 probes passed quality control for the 37 species of interest, with an average of 6.9±2.6 marker genes for each species (min 1 for *S. haemolyticus*, max 10 for several species) and 2.0±1.6 probes per gene (Table 1).

A characterization of these marker genes based on high-level Cluster of Orthologous Groups of proteins (COGs) [32] included selections from all top-level pathways (Figure S1). BactoChip-targeted genes were under-enriched for uncharacterized proteins not matching any COG category, and conversely overrepresented “replication, recombination and repair” genes (COG L) compared to the total distribution in available genomes (14% against 4%). These two functional categories varied most from the background of all genomes, with most other functional categories demonstrating an even selection among BactoChip probes. Additional analysis with targeted functional databases determined that 19.97% of the markers are virulence factors (as defined by VFDB 2012 [33], [34]), and 4.99% are involved in antibiotic resistance (as define by ARDB [35]). This large fraction of pathogenicity-related genes is consistent with the pathogenic phenotype of most of the targeted species in the BactoChip and with the species-specificity of virulence genes.

### The BactoChip Correctly Identifies 37 Clinically Relevant Bacteria at the Species Level

**Figure 2:**
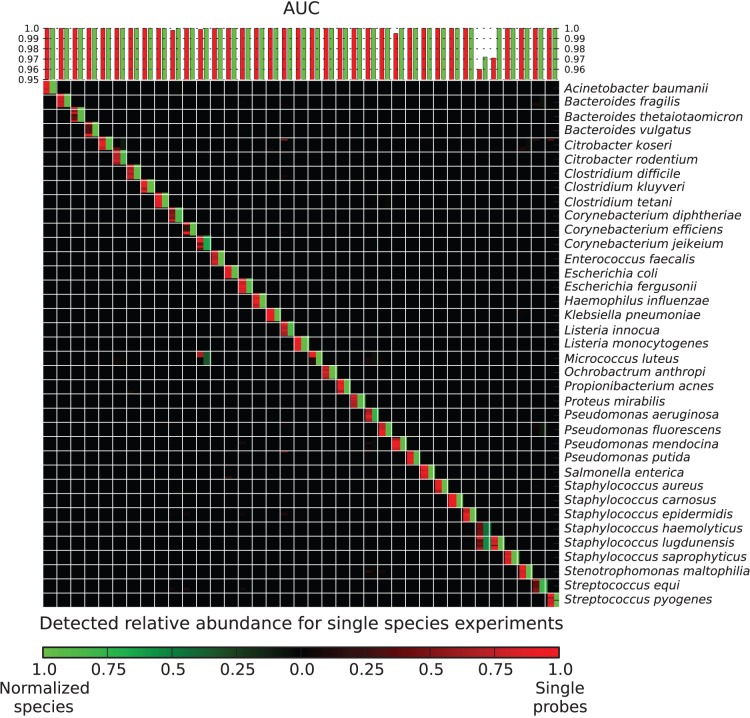
Accurate detection of individual bacterial species using the BactoChip. Bar plot indicates the Areas Under the receiver operating characteristic Curves (AUCs) for detection of 37 individual target species. Heatmap shows intensities of individual probes (red) and postprocessed aggregate species quantification (green). Almost all tested species (94.6%) were identified unequivocally by the specifically designed oligonucleotide probes, both based on raw data (see diagonal) and by comparing against the microbial gold standard (all AUC values >0.96).

As an initial validation, microbial samples each containing DNA from a single bacterial isolate were prepared for 37 randomly chosen species with available strains, from among the 54 bacterial species included during array design. Genomic DNA was either purchased from the german collection of microorganisms and cell cultures (DSMZ, from Deutsche Sammlung von Mikroorganismen und Zellkulturen GmbH) (Leibniz Institute DSMZ, Braunschweig, Germany) or extracted from microbial isolates; these were obtained from LGC Standards American Type Culture Collection (ATCC) (Manassas, VA, USA) and DSMZ catalog strains as vacuum dried cultures or from the Hospital Santa Chiara (Trento, Italy) as actively growing cells. The identity of hospital isolates from this study was confirmed by 16S rRNA gene sequencing (Table S1). Both types of microbial samples were prepared for the BactoChip by chemical labeling with the Universal Linkage System (ULS) linked to the Cy3 fluorophore (Agilent Technologies, Santa Clara, CA, USA). As shown in Figure 2, most individual BactoChip probes (red) and nearly all postprocessed aggregate species abundances (green) were determined to provide accurate assessments of microbial species.

Based on per-sample Areas Under the receiver operating characteristic Curves (AUCs) for probe and aggregate intensities, almost all species were perfectly identified. In all but five of 37 cases (13.5%), the preprocessed and normalized probes with the highest intensities were exactly and only the probes designed for the sample’s target species. After computational postprocessing, 36 of 37 test species (97.3%) were perfectly identified (AUC values higher than 0.99). This includes cases in which the BactoChip continued to perform well even when a finished genome for the specific isolate strain present in the sample was unavailable (e.g. *Acinetobacter baumanii*, *Klebsiella pneumonia,* and *Staphylococcus carnosus*).

Of the small fraction of individually malfunctioning probes, only three represented false negatives, i.e. probes with no or low signal for the intended target species, specifically *Corynebacterium efficiens, Micrococcus luteus* and *Pseudomonas mendocina*. In these cases, either the target gene was not in the core genome of the tested strain as expected, or the specific 60-mer sequences targeted by these probes (and by probes removed during standard preprocessing) were affected by polymorphisms relative to the sequenced reference genomes. Likewise, two sets of false positive probes affected our validations (Figure 2): 1 probe for *Corynebacterium diphtheriae* and *Corynebacterium jeikeium* and 20 for *Staphylococcus haemolyticus*. These false positives, together with completely inactive probes (never above the 95^th^ percentile and removed during preprocessing), arose due to the limited number of complete genomes available at the time of chip design, leading to an overestimation of genes’ uniqueness to each species (e.g. only 1 genome available for *Listeria innocua*). One of the most notable of these, a probe designed for *C. diphtheriae* that activated for *Propionibacterium acnes*, may be due to a horizontal gene transfer event that did not occur in the sequenced *P. acnes* strain.

In all but one of these five examples, our overall microarray design prevented these outliers from impacting species detection, as it incorporates several probes per gene and several genes per species. In fact, after computational postprocessing that included inactive probe removal, gene copy number calibration, and aggregation of all probes in each species, only one species (*Staphylococcus haemolyticus*) remained below the maximum achievable AUC. Overall, this extensive validation of the BactoChip with single bacteria selected from more than two thirds of the chip’s current targets demonstrated high accuracy in identifying the individual bacterial species.

### The Abundances of Multiple Congeneric Bacterial Species are Accurately Estimated within Mixed Samples

**Figure 3:**
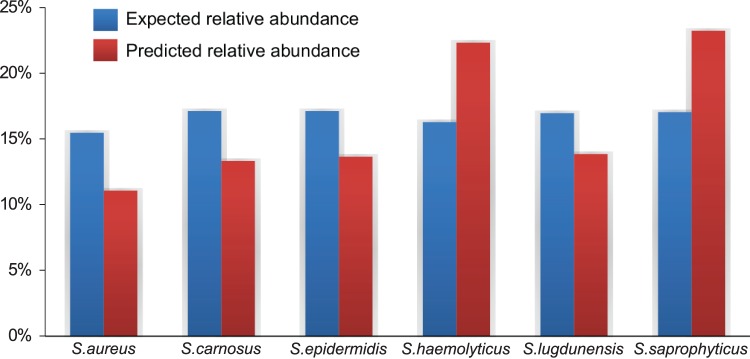
Quantification of the relative abundances of multiple species from the same genus contained within a single sample. True versus detected relative abundances for each of 6 *Staphylococcus* species are shown in blue (gold standard) and red (inferred), respectively. Even DNA relative abundances were targeted, with true experimental abundances of cell copy numbers varying due to differences in genome size. Mean Squared Error (MSE) of relative abundance over all predictions was below 0.0019.

We next assessed the BactoChip’s ability to additionally discriminate multiple mixed bacterial species within the same genus. This is of particular interest for genera such as *Staphylococcus* in which some species may be pathogenic and others commensal; these are often poorly resolved by single 16S genes [36], [37]. This genus contains extremely diverse species found throughout the human microbiome [38] and includes organisms of clinical interest such as *S. aureus*. We thus tested the BactoChip on 6 species from the clinically relevant staphylococci comprising *S. aureus, S. carnosus, S. epidermidis, S. haemolyticus*, *S. lugdunensis*, and *S. saprophyticus*. Hybridization of this mixed sample on the BactoChip resulted in all six species being correctly identified (Figure 3).

When considering multiples species in a single sample, it is important to identify both which species are present as well as their relative abundances. Although some pathogens can cause infection even when present at very low concentration, complex diseases such as obesity or inflammatory bowel disease have been connected with quantitative shifts in commensal bacterial community composition [39], [40]. After probe calibration and computational postprocessing (see Methods), all relative abundances of organisms were correctly estimated by the BactoChip within an average deviation of 24%. Thus even within the genus *Staphylococcus* along, mixed species could be measured reliably. The proposed microarray was both able to accurately differentiate among closely related species within a single sample and to determine their relative abundances quantitatively to a high degree of accuracy.

### The BactoChip Enables Species-level Abundance Quantification within Microbial Communities

We also extended our validation of samples containing multiple species to mixed communities of up to 15 different organisms belonging to 12 genera. Complex microbial communities are of increasing translational interest for analyses of the human microbiome [41], which can require the simultaneous quantification of up to hundreds of bacterial taxa. As a more modest evaluation of this initial version of the BactoChip, we designed two synthetic bacterial communities comprising 9 and 15 different species, respectively (Table S1). Each synthetic community was prepared by mixing defined microbial DNA species, as quantified by Nanodrop ND1000; the first community comprised 9 species from 8 different genera, whereas the second community 15 species from 12 different genera. We tested four versions of each community comprising high (63.3–146.8 ng) and low (6.3–14.7 ng) microbial DNA concentrations at both evenly distributed and at staggered concentrations (up to 30-fold within-sample relative abundance differences among species).

**Figure 4:**
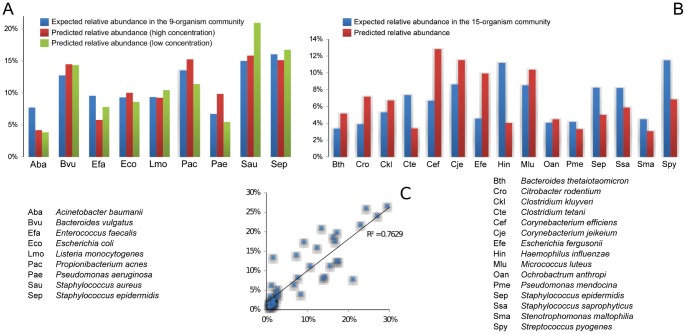
Accurate determination of microbial community composition of up to 15 species at even and staggered abundances. (A) Bar plots show true (blue) versus predicted (red and green) relative abundances for a 9-species bacterial community with evenly distributed abundances and high or low DNA concentrations. MSE of relative abundance over all predictions was below 0.0006. (B) True (blue) versus predicted (red) abundances for a 15-species evenly distributed community, total MSE <0.0013. (C) Evaluation of the BactoChip’s overall quantitation of relative microbial abundances in all four staggered communities. Each point represents the predicted vs. true relative abundance for one species in one experiment, with total R^2^>0.75.

The BactoChip was able to correctly identify bacterial presence and absence within these two communities with species-level specificity (Figure 4, Figure S2). Quantitatively, the estimated relative abundances closely approximated the expected community compositions. In the smaller of the two communities, evenly distributed abundance estimations deviated from reference values by less than 15% in half of the cases, the worst result being an estimation of 3.9% of *Acinetobacter baumanii* against an expected value of 7.7%. The staggered abundance setting achieved similar results, with an overall correlation of 0.97 (p<10^−10^) of predicted values with reference. Even in the larger community, microbial composition was correctly identified in each case, with only two outliers (*Stenotrophomonas maltophilia* and *Streptococcus pyogenes*) in a formulation of the community with staggered abundances; these were likely due to a swapped sample label and were not considered in subsequent experiments, as available resources precluded the possibility of proving or disproving a mislabeling. For the evenly distributed formulation of this community, all relative abundance estimations were within at most two-fold of the expected value.

The BactoChip probe design and computational calibration can thus continue to be developed to enable increasingly accurate relative abundance quantification in more diverse communities such as the human microbiome, although it would already be appropriate for reduced-complexity samples (e.g. hospital surfaces or clinical isolates [42]. However, we did observe only a small effect of low (6.3–14.7 ng) microbial DNA concentration (root mean squared error of 0.022 versus 0.027 for the first mix), which would negatively impact the high-throughput sequencing approaches currently applicable to such diverse communities. Likewise, these data confirm the BactoChip’s ability to detect microbes as rare as 3% of a community, and its lower limit of detection reached approximately two orders of magnitude smaller (bounds lowered with the saliva microbiome samples detailed below). Overall, in combination with the single-species and single-genus experiments above, these evaluations show that the BactoChip achieved near-perfect detection of bacterial species, discriminated among species from the same genus, and could be applied to microbiomes of modest complexity and with uneven species concentrations to reliably estimate microbial community composition.

### The BactoChip Accurately Detects Changes in Absolute Microbial Abundance in a Comparative Setting

Unlike sequencing-based approaches, which are limited to assessing relative microbial abundances, the Bactochip proved capable of accurately determining differences in absolute levels of microbial abundance among samples. This proved true even of species-level fold-change differences in multi-organism communities assayed on different arrays. Four formulations of the 9-species community described above were assayed, two at even abundance distributions and two staggered, both with different absolute DNA concentrations. When contrasting absolute probe intensities for organisms in the high and low concentration versions, as well as the two staggered versions of this community, we detected a fold change higher than 2.5 for all and only the expected species (Table 2). The same level of detection held for the larger community as well (Table 2), confirming that the BactoChip’s ability to qualitatively assess absolute microbial load in a comparative setting was robust within medium-complexity microbial communities.

**Table 2:** Detection of changes in absolute microbial load among samples.

9-organism community		Predicted fold-change	
		>2.5	<2.5
**Difference absolute abundance**	**Yes**	36	0
	**No**	0	18
**15-organism community**		**Predicted fold-change**	
		**>2.5**	**<2.5**
**Difference absolute abundance**	**Yes**	23	0
	**No**	3	13

Evaluation of the predicted difference in absolute microbial abundance (detected fold change larger then 2.5) with respect to the expected variations for (A) the 9-species community and (B) the 15-species community.

These fold change estimations between communities were performed on absolute probe intensities, which in turn serve as a proxy for absolute microbial concentrations. In a setting such as this in which at least one between-arrays comparison is possible, we can thus provide approximate information on both relative and absolute microbial abundances within communities. This has yet proven infeasible with other approaches such as shotgun or 16S-rRNA sequencing, which provide only relative abundances, thus limiting the interpretation of changes in community composition among samples [43]. For example, a sequencing-based comparison between the high and low concentration formulations of our first community would produce no detectable differences after conversion to relative abundances, despite a 10-fold variation in number of microbial cells. Although further calibration will be needed to make such determinations of absolute abundance more quantitative, this evaluation demonstrates that the BactoChip is capable of comparatively detecting absolute microbial abundances in a manner not possible using high-throughput sequencing, making it a flexible alternative for microbial assessment in case-control investigations in addition to community composition profiling.

### The BactoChip Detects Native Species and Positive Control Organisms in the Human Oral Microbiome

**Figure 5:**
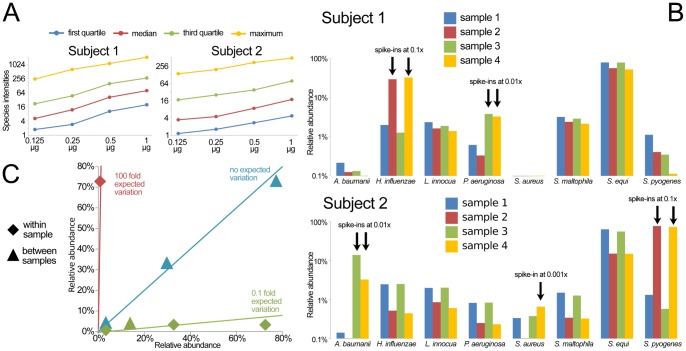
Application of the BactoChip to the oral microbiome detects native Streptococcus spp. and correctly identified the introduced spike-in species. A) Species-level averaged intensities linearly correlate with the total measured DNA in the range 0.125–1.000 µg. B) BactoChip detected the presence of *Streptococcus equi* in the saliva samples of both subjects at abundances higher than 50%. The introduced spike-ins for five different species at different abundances (from about 0.1% to 10% of the total community) were successfully identified in all cases with accurate distinction between different *Streptococcus* species. Only the species with a relative abundance greater than 1.5% in at least one sample are reported. C) Quantitative evaluation of the predictions for the introduce spike-ins looking in terms of fold change between the abundances within and between samples. All comparison showed strong consistency with the expected fold changes.

Finally, we applied the BactoChip to profile the oral microbiomes of two healthy subjects. Saliva samples from two subjects were extracted and hybridized with and without spike-ins of one to three microbes present at relative abundances ranging from 10% to 0.1% of total DNA. The chip’s quantification of species-level hybridization intensities relative to the total microbial load (from 0.125 µg to 1.000 µg) was extremely linear across the assessed concentration ranges (Figure 5A). This allowed us to select a target of 0.250 µg of microbial DNA per sample for further experiments, which is thus also a reasonable recommendation for future microbial community studies on the BactoChip.

For both tested subjects, the BactoChip detected the native presence of *Streptococcus equi* (>90%), *Stenotrophomonas maltophilia*, *Haemophylus influenzae*, and *Listeria innocua* (Figure 5B). As the BactoChip was designed to address almost exclusively pathogenic microbes, most genera typically found in the healthy saliva microbiome (as established by 16S sequencing on a large cohort [44]) are in fact not currently targeted by the BactoChip, both by design and due to the reduced panel of originally available sequenced genomes. We thus augmented both subjects’ samples with specific species over a range of concentrations with respect to the original total bacterial load, which were both detected and correctly quantified in all nine cases (Figure 5B). The correct detection of *Streptococcus pyogenes* independently from other *Streptococcus* spp. (specifically the phylogenetically close *S. equi*) is of particular interest, as it demonstrates the species-specificity of the BactoChip and its robustness to cross-hybridization.

We compared the estimated abundances of spikes-in within and across samples, and the expected fold change values were observed for all eight intra-subject comparisons (Figure 5C). This within-study normalization is necessary in this case since direct quantitation of single spikes-in is precluded due to the unknown fraction of the microbial community comprised by non-target organisms. These organisms did also highlight, however, the BactoChip’s ability to detect microbes as rare as 0.1% of the total community at over a 100-fold dynamic range (for example, *S. aureus* at 0.001× concentration, Figures 5B–C).

## Discussion

Defining the composition of bacterial communities is important for basic research, for microbial epidemiology, and for clinical investigations. In order to realize both the throughput and specificity of sequence-based techniques for microbial identification, we created the BactoChip, an oligonucleotide microarray targeting species-specific marker genes. In this initial study, the BactoChip proved able to identify 37 species from 19 genera with near-perfect accuracy, even in the presence of multiple species from the same genus (e.g. for the clinically relevant *Staphylococcus* genus). Moreover, in 7 complex communities of up to 15 organisms at 10-fold different relative abundances, the BactoChip proved able to quantify relative abundances with high accuracy (ρ = 0.97, p<10^−10^). While these synthetic communities are simpler than the thousands of organisms of the commensal human microbiota [32], they serve as a proof-of-principle that the BactoChip can scale from single pathogen detection to whole-community characterization in a clinically accessible manner.

Previous work on microarrays for microbial quantification has focused on the universal 16S rRNA gene, while the BactoChip employs a novel *in silico*-driven selection of unique species-specific marker genes. The 16S rRNA marker has a long history in DNA-sequencing based microbial quantification, but even there poses substantial challenges due to necessary amplification steps, differing copy numbers among organisms, and, critically, a frequent lack of resolution below the genus level [45]. Its use on microarrays adds the complexity of cross-hybridization, as the 16S sequence is, by definition as a “universal” marker, quite well-conserved [46]. Conversely, we selected species-specific genes guaranteed to be unique to targets of interest, within the limits of currently available finished microbial genomes (now over 1,300), which permits both greater detection accuracy and the opportunity to investigate the evolutionary history of the selected genes.

Considerable variability was observed in the number of unique sequences for each species, ranging from about 50 in *E. coli* (given its genomic closeness to *Shigella*) to almost 2,000 in *B. thetaiotaomicron*. This is not surprising, given the high stringency of the conservation threshold (99%) chosen for maximizing sequence conservation in this setting, the influences of intra-genus and intra-species diversity, the number of available genomes for the species considered, and the different rates of horizontal gene transfer among clades [47]. In some cases, few marker genes were available due to the large overlap of a species’ pan-genome with the corresponding genus pan-genome (e.g. *Listeria monocytogenes*) or its overall sequence diversity (e.g. *Stenotrophomonas maltophilia* [48]). In other cases, species-level core genes were readily available, but they were only infrequently determined to be unique, as a consequence of horizontal gene transfer events or of strong conservation (e.g. the Enterobacteriaceae [49]). These features were detected by our computational pipeline but they do not impact the BactoChip itself.

The characteristics of the BactoChip compare favorably to those of existing methods for clinically-oriented bacterial identification and quantification. While no high-throughput method can compete with the current cost or standardization of single-organism culture or amplification techniques, our approach has clear advantages when fastidious microbes must be targeted. Likewise it scales to quantify relative abundances of dozens or potentially hundreds of organisms simultaneously, which is impractical for culture- or PCR-based techniques and currently unfeasible in clinical practice using high-throughput sequencing. Finally, these microarray results themselves are consistent with a smaller study reporting identification of bacterial species specifically within the Enterobacteriaceae [49]. Since our approach is scalable and limited only by the number and diversity of available microbial genomes, it could be expanded from the species to the strain level or used to target non-bacterial microbes (e.g. Fungi or Archaea [50]). Moreover, this microarray platform seamlessly permits the incorporation of functional marker sequences in addition to taxonomic markers; for example, we anticipate targeting a set of virulence factor genes in the next revision, thus allowing the estimation of a sample’s pathogenic or antibiotic potential in addition to its composition.

It is interesting to note that, while the BactoChip was particularly useful in the clinically-relevant task of high-throughput species-specific bacterial detection, it also showed promise in fully quantifying relative abundances of microbes in mixed populations. We piloted here 2 communities with 7 different configurations, using both synthetically even and more physiological staggered organismal abundances, with this first iteration of the BactoChip achieving high precision with up to 15 organisms. In all of these communities, our taxonomic marker genes proved to remain specific down to the species level, with few errors in identification and most imprecisions arising instead in the quantification of relative abundance. Finally, BactoChip experiments on the human saliva microbiome from two healthy subjects confirmed its applicability to true high-complexity samples and suggested a limit of detection at least as low as 0.1%.

Since 16S-based microarrays such as the PhyloChip, and HITChip have proven quite successful in quantifying genus-level relative abundances [18], [51], we anticipate that we will be able to further improve our precision in this task at the species-level using targeted probe design, biophysical modeling for normalization, and additional chip-level positive and negative controls in future iterations of the microarray. In conclusion, this study’s novel combination of *in silico* marker gene selection with microbial species detection and quantification by microarray shows the feasibility of the approach. We believe these tools will pave the way for the further development of reliable and inexpensive microarray-based methods for culture-independent species-level microbial screening, particularly in clinical settings.

## Materials and Methods

The design and testing of the BactoChip consisted of two major steps. In the first, we identified *in silico* species-specific genetic markers and designed oligonucleotide probes for these genes. The second step involved experimental assessment of this microarray design, including the hybridization of DNA from reference bacterial strains and clinical isolates.

### *In silico* Design

#### Identification of species-specific marker genes

The coding sequences of 186 annotated complete bacterial genomes comprising 54 clinically relevant species were downloaded from the National Centre for Biotechnology Information microbial database (http://www.ncbi.nlm.nih.gov/genomes/lproks.cgi) as of June 2010. We identified core genes with >99% similarity, if not otherwise stated, within all complete genomes available for each of the 54 species. This is a particularly stringent threshold adopted in order to identify the most conserved genes at species level. We then selected unique genes for each bacterial species, i.e. core genes with no blastn hits to genomes outside the target species. Few species (e.g. *Listeria monocytogenes*) showed low enough sequence conservation to produce fewer than 10 core or unique genes; in these cases, the similarity percentage required for core gene selection was reduced to 80% to increase the number of candidates (otherwise prioritized identically with respect to intra-species conservation). Since this had the potential to increase the false negative rate due to reduced affinity in DNA probe hybridization, we chose multiple marker genes for such species to counteract potential false assignments due to higher false negative rate.

We functionally profiled the unique markers by mapping them against COGs [32] sequences using BLASTP (e-value threshold <1e–5 with best hit policy). The resulting distribution of top-level functional categories was compared to that of a baseline obtained by profiling all Integrated Microbial Genomes database [52] genes. Similarly, we tested the presence of virulence factors and antibiotic resistance genes using comparable mappings to the VFDB 2012 [33],[34] and ARDB [35] databases respectively.

#### Probe selection for unique genes

Oligonucleotide probes were designed using the AlleleID software version 7.01 (Premier Biosoft Int., Palo Alto, CA, USA). The probes consisted of 57- to 60–mers with similar physicochemical parameters (GC>40%, T_m_ = 73°C±5°C). Probes were selected according to the number of hits with 100% identity within the target bacterial species. To ensure reliable identification of the intended bacteria, probes targeting an average of 6.9±2.6 unique-genes were designed for each bacterial species (see Table 1 for probes passing subsequent quality control). The full set of probe IDs, sequences and target unique-gene are shown at ArrayExpress database (www.ebi.ac.uk/arrayexpress) with accession number E-MEXP-3420.

#### Microarray design

Custom microarrays with the designed oligonucleotide probes were purchased from Agilent-Technologies (Santa Clara, CA, USA) using the “custom high-definition Agilent DNA Comparative Genomic Hybridization arrays 8×15 k”. This format is suitable to this study’s goal of microbiome-wide profiling of DNA copy number changes associated with microbial agents. The custom microarray comprised 2,094 marker gene probes, 87 internal controls from Agilent or added during design, 140 Agilent negative controls, 49 Agilent dark corners and 2898 additional probes targeting genes of functional interest. For each marker gene probe three technical replicates were randomly distributed on the chip (see ArrayExpress database, experiment E-MEXP-3420).

#### Bacterial and clinical sample collection

Bacterial collection included 37 target species belonging to 19 genera, chosen for their high prevalence in hospital settings and the availability of annotated complete genomic sequences as of June 2010. 1–2 off-target species were selected for assessment within each bacterial genus. Strains for each species were purchased when available as vacuum dried culture or DNA from the LGC Standards ATCC and the Leibniz Institute DSMZ. Others were collected as actively growing cultures clonally isolated on blood-agar media from clinical specimens at the Microbiology and Virology Unit (Santa Chiara Hospital, Trento, Italy). Gold standard strains used for experimental evaluation of the microarray are described in the supplemental data (Table S1). Clinical isolates were identified at the hospital by routine culture-based methods and confirmed by 16S rRNA gene sequencing.

Clinical samples were obtained from 2 healthy subjects who received oral hygiene instructions and gave their informed consent. Up to 15 mL saliva samples were collected from the 2 subjects by non-stimulated drooling into sterile plastic tubes. Samples were processed within 2 hours from the collection time as previously described [53].

### Experimental Design

#### Total DNA isolation

High-purity total bacterial DNA was extracted with silica-based DNA purification QIAGEN DNeasy Blood&Tissue kit (QIAGEN GmbH, Hilden, Germany) and eluted in 200 µL sterile distilled water. DNA quality was assessed by agarose gel electrophoresis after staining with ethidium bromide, whereas DNA purity and concentration was determined by measuring absorbance at 230, 260, and 280 nm with the Nanodrop ND1000 (NanoDrop products, Wilmington, DE, USA). Total DNA from saliva samples was extracted as previously described [52] and purified with the QIAGEN DNeasy Blood&Tissue kit as for pure bacterial cultures.

#### Quantification of bacterial DNA in saliva samples

The bacterial load in saliva samples was determined by amplification and detection of the 16S rRNA gene by real-time PCR, performed with the CFX96 real-time PCR detection system (Bio-Rad Laboratories S.r.l., Milan, Italy), using optical grade 96-well plates. The PCR reaction was performed in a 25 µL final volume using 2.5 µL of 2X QuantiTect SYBR Green mix (Qiagen S.r.l., Milan, Italy), 3 µL template DNA and 0.5 uM of each of the universal primers: forward primer, 5′-TCCTACGGGAGGCAGCAGT-3′ and reverse primer, 5′-GGACTACCAGGGTATCTAATCCTGTT-3′ [54]. The PCR protocol comprised an initial denaturation at 95°C for 10 min, 40 cycles of 95°C 15 sec, 60°C 20 sec, 72°C 40 sec and a melting curve analysis from 65°C to 95°C and fluorophore detection at last step of each cycle. *Escherichia coli* DNA from ATCC25922 strain was used as a standard for determining the absolute quantification of saliva samples with or without a spiked-in bacterial DNA at known concentration. Quantified values for saliva samples were corrected taking in account the difference in amplification efficiency between *E. coli* known quantity and the same spiked in saliva samples.

#### DNA labeling

Total DNA was fragmented for 3 min at 95°C and chemically labelled with the ULS linked to the Cy3 fluorophore, as described in the Genomic DNA analysis ULS Labeling Kit manual (Agilent Technologies, Santa Clara, CA, USA).

#### Microarray hybridization

Labelled samples, resuspended in hybridization cocktail, were hybridized to the custom microarray according to the manufacturer’s instruction manual (Agilent Technologies, Santa Clara, CA, USA). Microarrays were tested with single or multiple labelled targets. An overview of all experiments performed is presented as supplemental data (Table S1 and ArrayExpress database, accession number E-MEXP-3420).

#### Image acquisition and data processing

Images were acquired with the DNA Microarray Scanner G2505C and the ScanControl software; data were extracted with the Feature Extraction software v10.7 using the Agilent one-colour gene expression protocol GE1_107_Sep09 (Agilent Technologies, Santa Clara, CA, USA). Raw and processed data are provided at ArrayExpress database, experiment E-MEXP-3420.

#### Computational postprocessing

Probe signal intensities were determined by subtracting the local background values (as estimated by the Agilent platform) from the per-sample median. Each probe’s three technical replicates were then averaged and a robust average (obtained removing values above the 95^th^ percentile and below the 5^th^ percentile) of all probes in the same species was performed. Species relative abundances were then estimated by relativizing this species average with respect to the sum of all averages. Unsupervised probe selection was performed by removing probes never showing signal above the 98^th^ percentile (false negative removal) or always above median in more than 75% of samples (aspecific false positive removal). Calibration for multi-species experiments was performed using single-species experiments first by normalizing each probe’s signal with respect to the concentration of genomes in the sample, estimated by transforming DNA concentrations to genome concentrations by dividing by the average genome length of sequenced strains within each species. Finally, positive probes below a 100x fold change with respect to the median of the negative probe distribution were removed for noise reduction.

## Supporting Information

Figure S1 **BactoChip markers represent all high-level functional areas within available genomes.** Yellow bars represent the fraction of BactoChip marker genes in each top-level functional category from the COG database, red the fractions from all genomes included in the Integrated Microbial Genomes database is also reported as a reference background distribution. Most fractions are comparable, with the BactoChip underenriched for uncharacterized proteins and overenriched for housekeeping DNA maintenance and structural proteins.
(EPS)

Figure S2 **Quantification of composition of 9-species and 15-species full communities, in staggered concentrations.** Bar plots show real versus predicted relative abundances in red and blue, respectively.
(EPS)

Table S1 **List of experiments performed in this study.** The 61 experiments on 37 species and saliva samples are listed here, with specifications on the experiment ID, the bacterial species tested and its strain ID, supply format and collection source reference. In the last two columns, the DNA quantity, as estimated by Nanodrop ND1000, and corresponding array ID are provided.
(DOCX)
