# Supplementary material for: Simultaneous Quantification of Multiple Bacteria by the BactoChip Microarray Designed to Target Species-Specific Marker Genes
Source: PLoS One. 2013 Feb 11;8(2):e55764. doi: 10.1371/journal.pone.0055764 (PMC3569451; doi:10.1371/journal.pone.0055764)
Supplement: Table S1 — List of experiments performed in this study. The 61 experiments on 37 species and saliva samples are listed here, with specifications on the experiment ID, the bacterial species tested and its strain ID, supply format and collection source reference. In the last two columns, the DNA quantity, as estimated by Nanodrop ND1000, and corresponding array ID are provided. (DOCX) [file pone.0055764.s003.docx]

**Table S1. List of experiments performed in this study.**

| Exp ID | Species | Strain ID | Supplied as | Reference | Quantity (ng) | Array ID |
| --- | --- | --- | --- | --- | --- | --- |
| 1 | Acinetobacter baumanii | ATCC 19606 | vacuum dried culture | a | 70.5 | no6_1.1 |
| 2 | Bacteroides fragilis | ATCC 25285 | vacuum dried culture | a | 69.6 | no4_1.1 |
| 3 | Bacteroides thetaiotaomicron | DSM 2079 T | DNA | b | 80.5 | no4_1.3 |
| 4 | Bacteroides vulgatus | DSM 1447 T | DNA | b | 146.8 | no4_1.2 |
| 5 | Citrobacter koseri | AB216 | actively growing culture | this study | 93.0 | no1_1.3 |
| 6 | Citrobacter rodentium | DSM 16636 T | vacuum dried culture | b | 274.0 | no1_1.4 |
| 7 | Clostridium difficile | DSM 1296 T | vacuum dried culture | b | 249.8 | no6_1.4 |
| 8 | Clostridium kluyveri | DSM 555 T | DNA | b | 978.7 | no3_1.2 |
| 9 | Clostridium tetani | DSM 11744 | DNA | b | 98.7 | no3_1.3 |
| 10 | Corynebacterium diphtheriae | DSM 43988 | vacuum dried culture | b | 84.9 | no6_1.3 |
| 11 | Corynebacterium efficiens | DSM 44549 T | vacuum dried culture | b | 132.0 | no5_2.3 |
| 12 | Corynebacterium jeikeum | DSM 7171 T | vacuum dried culture | b | 117.2 | no6_2.2 |
| 13 | Enterococcus faecalis | ATCC 29212 | vacuum dried culture | a | 66.9 | no5_1.3 |
| 14 | Escherichia coli | ATCC 25922 | vacuum dried culture | a | 105.4 | no3_2.1 |
| 15 | Escherichia fergusonii | DSM 13698 T | vacuum dried culture | b | 71.1 | no3_2.2 |
| 16 | Haemophilus influenzae | ATCC 49766 | vacuum dried culture | a | 42.9 | no4_2.3 |
| 17 | Klebsiella pneumoniae | ATCC 33495 | vacuum dried culture | a | 45.5 | no5_1.1 |
| 18 | Listeria innocua | DSM 20649 T | vacuum dried culture | b | 98.4 | no2_2.1 |
| 19 | Listeria monocytogenes | ATCC 19115 | vacuum dried culture | a | 101.3 | no2_2.2 |
| 20 | Micrococcus luteus | DSM 20030 T | vacuum dried culture | b | 102.3 | no1_2.4 |
| 21 | Ochrobactrum anthropi | DSM 6882 T | vacuum dried culture | b | 100.6 | no9_1.1 |
| 22 | Propionibacterium acnes | DSM 1897 T | DNA | b | 99.7 | no2_2.4 |
| 23 | Proteus mirabilis | ATCC 7002 | vacuum dried culture | a | 80.6 | no2_1.2 |
| 24 | Pseudomonas aeruginosa | ATCC 27853 | vacuum dried culture | a | 37.5 | no2_2.3 |
| 25 | Pseudomonas fluorescens | DSM 4358 | vacuum dried culture | b | 118.7 | no1_2.2 |
| 26 | Pseudomonas mendocina | DSM 50017 T | vacuum dried culture | b | 96.4 | no8_1.2 |
| 27 | Pseudomonas putida | DSM 291 T | vacuum dried culture | b | 85.7 | no1_2.3 |
| 28 | Salmonella enterica | ATCC 14028 | vacuum dried culture | a | 99.6 | no8_1.3 |
| 29 | Staphylococcus aureus | ATCC 29213 | vacuum dried culture | a | 98.8 | no3_2.3 |
| 30 | Staphylococcus carnosus | DSM 20501 T | vacuum dried culture | b | 100.0 | no10_1.1 |
| 31 | Staphylococcus epidermidis | AB45 | actively growing culture | this study | 95.5 | no3_2.4 |
| 32 | Staphylococcus haemolyticus | AB189 | actively growing culture | this study | 100.0 | no10_1.2 |
| 33 | Staphylococcus lugdunensis | AB312 | actively growing culture | this study | 100.0 | no10_1.3 |
| 34 | Staphylococcus saprophyticus | AB26 | actively growing culture | This study | 100.0 | no10_1.4 |
| 35 | Stenotrophomonas maltophilia | ATCC 49130 | vacuum dried culture | b | 97.5 | no1_1.2 |
| 36 | Streptococcus equi | DSM 20561 T | vacuum dried culture |  | 97.7 | no2_1.3 |
| 37 | Streptococcus pyogenes | AB255 | actively growing culture | this study | 100.5 | no9_1.2 |
| 38 | Staphylococcus aureus | ATCC 29213 | vacuum dried culture | b | 100.0 | no10_2.4 |
| 38 | Staphylococcus epidermidis | AB45 | actively growing culture | this study | 100.0 | no10_2.4 |
| 38 | Staphylococcus carnosus | DSM 20501 T | vacuum dried culture | a | 100.0 | no10_2.4 |
| 38 | Staphylococcus haemolyticus | AB189 | actively growing culture | this study | 100.0 | no10_2.4 |
| 38 | Staphylococcus lugdunensis | AB312 | actively growing culture | this study | 100.0 | no10_2.4 |
| 38 | Staphylococcus saprophyticus | AB26 | actively growing culture | this study | 100.0 | no10_2.4 |
| 39 | Acinetobacter baumanii | ATCC 19606 | vacuum dried culture | a | 70.5 | no8_2.1 |
| 39 | Bacteroides vulgatus | DSM 1447 T | vacuum dried culture | b | 146.8 | no8_2.1 |
| 39 | Enterococcus faecalis | ATCC 29212 | vacuum dried culture | a | 66.9 | no8_2.1 |
| 39 | Escherichia coli | ATCC 25922 | vacuum dried culture | a | 111.6 | no8_2.1 |
| 39 | Listeria monocytogenes | ATCC 19115 | vacuum dried culture | a | 63.3 | no8_2.1 |
| 39 | Propionibacterium acnes | DSM 1897 T | vacuum dried culture | b | 78.7 | no8_2.1 |
| 39 | Pseudomonas aeruginosa | ATCC 27853 | vacuum dried culture | a | 100.9 | no8_2.1 |
| 39 | Staphylococcus aureus | ATCC 29213 | vacuum dried culture | a | 98.8 | no8_2.1 |
| 39 | Staphylococcus epidermidis | AB45 | actively growing culture | this study | 95.5 | no8_2.1 |
| 40 | Acinetobacter baumanii | ATCC 19606 | vacuum dried culture | a | 7.0 | no8_2.2 |
| 40 | Bacteroides vulgatus | DSM 1447 T | vacuum dried culture | b | 14.7 | no8_2.2 |
| 40 | Enterococcus faecalis | ATCC 29212 | vacuum dried culture | a | 6.7 | no8_2.2 |
| 40 | Escherichia coli | ATCC 25922 | vacuum dried culture | a | 11.2 | no8_2.2 |
| 40 | Listeria monocytogenes | ATCC 19115 | vacuum dried culture | a | 6.3 | no8_2.2 |
| 40 | Propionibacterium acnes | DSM 1897 T | vacuum dried culture | b | 7.9 | no8_2.2 |
| 40 | Pseudomonas aeruginosa | ATCC 27853 | vacuum dried culture | a | 10.1 | no8_2.2 |
| 40 | Staphylococcus aureus | ATCC 29213 | vacuum dried culture | a | 9.9 | no8_2.2 |
| 40 | Staphylococcus epidermidis | AB45 | actively growing culture | this study | 9.5 | no8_2.2 |
| 41 | Acinetobacter baumanii | ATCC 19606 | vacuum dried culture | a | 70.5 | no8_2.3 |
| 41 | Bacteroides vulgatus | DSM 1447 T | vacuum dried culture | b | 14.7 | no8_2.3 |
| 41 | Enterococcus faecalis | ATCC 29212 | vacuum dried culture | a | 66.9 | no8_2.3 |
| 41 | Escherichia coli | ATCC 25922 | vacuum dried culture | a | 11.2 | no8_2.3 |
| 41 | Listeria monocytogenes | ATCC 19115 | vacuum dried culture | a | 63.3 | no8_2.3 |
| 41 | Propionibacterium acnes | DSM 1897 T | vacuum dried culture | b | 7.9 | no8_2.3 |
| 41 | Pseudomonas aeruginosa | ATCC 27853 | vacuum dried culture | a | 100.9 | no8_2.3 |
| 41 | Staphylococcus aureus | ATCC 29213 | vacuum dried culture | a | 9.9 | no8_2.3 |
| 41 | Staphylococcus epidermidis | AB45 | actively growing culture | this study | 95.5 | no8_2.3 |
| 42 | Acinetobacter baumanii | ATCC 19606 | vacuum dried culture | a | 7.0 | no8_2.4 |
| 42 | Bacteroides vulgatus | DSM 1447 T | vacuum dried culture | b | 146.8 | no8_2.4 |
| 42 | Enterococcus faecalis | ATCC 29212 | vacuum dried culture | a | 6.7 | no8_2.4 |
| 42 | Escherichia coli | ATCC 25922 | vacuum dried culture | a | 111.6 | no8_2.4 |
| 42 | Listeria monocytogenes | ATCC 19115 | vacuum dried culture | a | 6.3 | no8_2.4 |
| 42 | Propionibacterium acnes | DSM 1897 T | vacuum dried culture | b | 78.7 | no8_2.4 |
| 42 | Pseudomonas aeruginosa | ATCC 27853 | vacuum dried culture | a | 10.1 | no8_2.4 |
| 42 | Staphylococcus aureus | ATCC 29213 | vacuum dried culture | a | 98.8 | no8_2.4 |
| 42 | Staphylococcus epidermidis | AB45 | actively growing culture | this study | 9.5 | no8_2.4 |
| 43 | Bacteroides thetaiotaomicron | DSM 2079 T | DNA | b | 100.0 | no10_2.1 |
| 43 | Citrobacter rodentium | DSM 16636 T | vacuum dried culture | b | 100.0 | no10_2.1 |
| 43 | Clostridium kluyveri | DSM 555 T | DNA | b | 100.0 | no10_2.1 |
| 43 | Clostridium tetani | DSM 11744 | DNA | b | 100.0 | no10_2.1 |
| 43 | Corynebacterium efficiens | DSM 44549 T | vacuum dried culture | b | 100.0 | no10_2.1 |
| 43 | Corynebacterium jeikeium | DSM 7171 T | vacuum dried culture | b | 100.0 | no10_2.1 |
| 43 | Escherichia fergusonii | DSM 13698 T | vacuum dried culture | b | 100.0 | no10_2.1 |
| 43 | Haemophilus influenzae | ATCC 49766 | vacuum dried culture | a | 100.0 | no10_2.1 |
| 43 | Micrococcus luteus | DSM 20030 T | vacuum dried culture | b | 100.0 | no10_2.1 |
| 43 | Ochrobactrum anthropi | DSM 6882 T | vacuum dried culture | b | 100.0 | no10_2.1 |
| 43 | Pseudomonas mendocina | DSM 50017 T | vacuum dried culture | b | 100.0 | no10_2.1 |
| 43 | Staphylococcus epidermidis | AB45 | actively growing culture | this study | 100.0 | no10_2.1 |
| 43 | Staphylococcus saprophyticus | AB26 | actively growing culture | this study | 100.0 | no10_2.1 |
| 43 | Stenotrophomonas maltophilia | ATCC 49130 | vacuum dried culture | a | 100.0 | no10_2.1 |
| 43 | Streptococcus pyogenes | AB255 | actively growing culture | this study | 100.0 | no10_2.1 |
| 44 | Bacteroides thetaiotaomicron | DSM 2079 T | DNA | b | 10.0 | no10_2.2 |
| 44 | Citrobacter rodentium | DSM 16636 T | vacuum dried culture | b | 100.0 | no10_2.2 |
| 44 | Clostridium kluyveri | DSM 555 T | DNA | b | 10.0 | no10_2.2 |
| 44 | Clostridium tetani | DSM 11744 | DNA | b | 100.0 | no10_2.2 |
| 44 | Corynebacterium efficiens | DSM 44549 T | vacuum dried culture | b | 10.0 | no10_2.2 |
| 44 | Corynebacterium jeikeium | DSM 7171 T | vacuum dried culture | b | 100.0 | no10_2.2 |
| 44 | Escherichia fergusonii | DSM 13698 T | vacuum dried culture | b | 10.0 | no10_2.2 |
| 44 | Haemophilus influenzae | ATCC 49766 | vacuum dried culture | a | 100.0 | no10_2.2 |
| 44 | Micrococcus luteus | DSM 20030 T | vacuum dried culture | b | 10.0 | no10_2.2 |
| 44 | Ochrobactrum anthropi | DSM 6882 T | vacuum dried culture | b | 100.0 | no10_2.2 |
| 44 | Pseudomonas mendocina | DSM 50017 T | vacuum dried culture | b | 10.0 | no10_2.2 |
| 44 | Staphylococcus epidermidis | AB45 | actively growing culture | this study | 100.0 | no10_2.2 |
| 44 | Staphylococcus saprophyticus | AB26 | actively growing culture | this study | 10.0 | no10_2.2 |
| 44 | Stenotrophomonas maltophilia | ATCC 49130 | vacuum dried culture | a | 10.0 | no10_2.2 |
| 44 | Streptococcus pyogenes | AB255 | actively growing culture | this study | 100.0 | no10_2.2 |
| 45 | Bacteroides thetaiotaomicron | DSM 2079 T | DNA | b | 100.0 | no10_2.3 |
| 45 | Citrobacter rodentium | DSM 16636 T | vacuum dried culture | b | 10.0 | no10_2.3 |
| 45 | Clostridium kluyveri | DSM 555 T | DNA | b | 100.0 | no10_2.3 |
| 45 | Clostridium tetani | DSM 11744 | DNA | b | 10.0 | no10_2.3 |
| 45 | Corynebacterium efficiens | DSM 44549 T | vacuum dried culture | b | 100.0 | no10_2.3 |
| 45 | Corynebacterium jeikeium | DSM 7171 T | vacuum dried culture | b | 10.0 | no10_2.3 |
| 45 | Escherichia fergusonii | DSM 13698 T | vacuum dried culture | b | 100.0 | no10_2.3 |
| 45 | Haemophilus influenzae | ATCC 49766 | vacuum dried culture | a | 10.0 | no10_2.3 |
| 45 | Micrococcus luteus | DSM 20030 T | vacuum dried culture | b | 100.0 | no10_2.3 |
| 45 | Ochrobactrum anthropi | DSM 6882 T | vacuum dried culture | b | 10.0 | no10_2.3 |
| 45 | Pseudomonas mendocina | DSM 50017 T | vacuum dried culture | b | 100.0 | no10_2.3 |
| 45 | Staphylococcus epidermidis | AB45 | actively growing culture | this study | 10.0 | no10_2.3 |
| 45 | Staphylococcus saprophyticus | AB26 | actively growing culture | this study | 100.0 | no10_2.3 |
| 45 | Stenotrophomonas maltophilia | ATCC 49130 | vacuum dried culture | a | 100.0 | no10_2.3 |
| 45 | Streptococcus pyogenes | AB255 | actively growing culture | this study | 10.0 | no10_2.3 |
| 46 | Oral microbiome | n.d. | Saliva subject 1 | this study | 1000.0 | no12_1.1 |
| 47 | Oral microbiome | n.d. | Saliva subject 1 | this study | 500.0 | no12_1.2 |
| 48 | Oral microbiome | n.d. | Saliva subject 1 | this study | 250.0 | no12_1.3 |
| 49 | Oral microbiome | n.d. | Saliva subject 1 | this study | 125.0 | no12_1.4 |
| 50 | Oral microbiome | n.d. | Saliva subject 2 | this study | 1000.0 | no12_2.1 |
| 51 | Oral microbiome | n.d. | Saliva subject 2 | this study | 500.0 | no12_2.2 |
| 52 | Oral microbiome | n.d. | Saliva subject 2 | this study | 250.0 | no12_2.3 |
| 53 | Oral microbiome | n.d. | Saliva subject 2 | this study | 125.0 | no12_2.4 |
| 54 | Oral microbiome | n.d. | Saliva subject 1 | this study | 125.0 | no13_1.1 |
| 55 | Oral microbiome | n.d. | Saliva subject 1 | this study | 125.0 | no13_1.2 |
| 55 | Haemophilus influenzae | ATCC 49766 | vacuum dried culture | a | 12.5 | no13_1.2 |
| 56 | Oral microbiome | n.d. | Saliva subject 1 | this study | 125.0 | no13_1.3 |
| 56 | Pseudomonas aeruginosa | ATCC 27853 | vacuum dried culture | a | 1.25 | no13_1.3 |
| 57 | Oral microbiome | n.d. | Saliva subject 1 | this study | 125.0 | no13_1.4 |
| 57 | Haemophilus influenzae | ATCC 49766 | vacuum dried culture | a | 12.5 | no13_1.4 |
| 57 | Pseudomonas aeruginosa | ATCC 27853 | vacuum dried culture | a | 1.25 | no13_1.4 |
| 58 | Oral microbiome | n.d. | Saliva subject 2 | this study | 250.0 | no13_2.1 |
| 59 | Oral microbiome | n.d. | Saliva subject 2 | this study | 250.0 | no13_2.2 |
| 59 | Streptococcus pyogenes | AB255 | actively growing culture | this study | 25.0 | no13_2.2 |
| 60 | Oral microbiome | n.d. | Saliva subject 2 | this study | 250.0 | no13_2.3 |
| 60 | Acinetobacter baumanii | ATCC 19606 | vacuum dried culture | a | 2.5 | no13_2.3 |
| 61 | Oral microbiome | n.d. | Saliva subject 2 | this study | 250.0 | no13_2.4 |
| 61 | Streptococcus pyogenes | AB255 | actively growing culture | this study | 25.0 | no13_2.4 |
| 61 | Acinetobacter baumanii | ATCC19606 | vacuum dried cultures | a | 2.5 | no13_2.4 |
| 61 | Staphylococcus aureus | ATCC 29213 | vacuum dried cultures | a | 0.25 | no13_2.4 |
| ^a^http://www.lgcstandards-atcc.org/  ^b^http://www.dsmz.de/ | | | | | | |
